# Precoding Method Interference Management for Quasi-EVD Channel

**DOI:** 10.1155/2014/678578

**Published:** 2014-08-28

**Authors:** Wei Duan, Wei Song, Sang Seob Song, Moon Ho Lee

**Affiliations:** ^1^Division of Electronic and Information Engineering, Chonbuk National University, Chonju 561-756, Republic of Korea; ^2^College of Information Technology, Eastern Liaoning University, Dandong, 118003, China

## Abstract

The Cholesky decomposition-block diagonalization (CD-BD) interference alignment (IA) for a multiuser multiple input multiple output (MU-MIMO) relay system is proposed, which designs precoders for the multiple access channel (MAC) by employing the singular value decomposition (SVD) as well as the mean square error (MSE) detector for the broadcast Hermitian channel (BHC) taken advantage of in our design. Also, in our proposed CD-BD IA algorithm, the relaying function is made use to restructure the quasieigenvalue decomposition (quasi-EVD) equivalent channel. This approach used for the design of BD precoding matrix can significantly reduce the computational complexity and proposed algorithm can address several optimization criteria, which is achieved by designing the precoding matrices in two steps. In the first step, we use Cholesky decomposition to maximize the sum-of-rate (SR) with the minimum mean square error (MMSE) detection. In the next step, we optimize the system BER performance with the overlap of the row spaces spanned by the effective channel matrices of different users. By iterating the closed form of the solution, we are able not only to maximize the achievable sum-of-rate (ASR), but also to minimize the BER performance at a high signal-to-noise ratio (SNR) region.

## 1. Introduction

Recently, wireless relay networks which are capable of improving the power efficiency, as well as the network coverage, have been studied with a lot of interest because relaying transmission is a promising technique which can be applied to extend the coverage or increase the system capacity. The capacity achieved by a point-to-point MIMO network has been shown to increase linearly with the minimum number of transceiver's antennas [1, 2]. Therefore, by employing multiple antennas at the transmitter or the receiver, the system can significantly improve the transmission reliability.

If multiple antennas are applied at both the transmitter and receiver sides, the channel capacity can be enhanced linearly with the minimum number of transmit and receive antennas [3].

Relay precoder designs for such a system have been reported in [4–6]. The problem of designing optimal beamforming vectors for multicasting is hard in general, mainly due to its nonconvex nature. In [4], the authors propose a transceive precoding scheme at the relay node by using zero-forcing (ZF) and MMSE criteria with certain antenna configurations. The information theoretic capacity of the multiantenna multicasting channel is studied in [5] with a particular focus on the scaling of the capacity and achievable rates as the number of antennas and users approaches infinity. In [6], the authors develop one algorithm to compute the globally optimal beamforming matrix at the relay node and characterize the system capacity region.

Most of the works mentioned above assume the availability of perfect channel state information (CSI) at the relay node [7, 8]. In practice, the CSI available at the relay node is usually imperfect due to different factors such as estimation error, quantization, and feedback delay. Interference alignment (IA) is proposed to achieve the maximum degree of freedom (DOF) for the *K*-user interference channels [9]. It designs the signals transmitted by all users with perfect CSI in such a way that the interfering signals at each receiver fall into a reduced-dimensional subspace. In order to implement IA scheme in the slow fading environment, multiple channels can be used for multiple carriers or multiple antennas [10]. Since these resources are limited, IA scheme with time extension is still efficient to support multiple users. In the past decades, researches on information theory have been exploring the capacity regions of Gaussian interference channels [11, 12]. In the *K*-user interference channel, it is proved that the IA scheme can provide the following capacity for each user:

(1) $$\begin{array}{c} C_{\text{IA}}=\frac{K}{2}\log\left(\text{SNR}\right)+o\left(\log\left(\text{SNR}\right)\right). \end{array}$$

Thus, in high-SNR regime, the capacity scales linearly with the number of users.

In this paper, we consider the problem of jointly designing the precoders and the relay transformation matrix for a one-way relay MIMO relay system, where all nodes have multiples antennas. Our goal is to use BHC and BD precoding design to decouple MU-MIMO channel into a set of *K* parallel independent SU-MIMO channels and CD-BD algorithm to reduce the computational complexity. In particular, the leakage interference is minimized in order to achieve interference alignment. By iterating the closed-form solution and precoding design, we reach the maximum sum-of-rate capacity and better performance in BER as shown in simulations.

The organization of the paper is as follows: Section 2 describes a general system model for the *K*-pairs one-way relay system, the definition of quasi-EVD, and global CSI. In Section 3, we propose an iterative CD-BD algorithm and optimal precoder design. In Sections 4 and 5, we discuss the ASR, DOF, and computational complexity for efficient channel model. The simulation results are presented to show the good performance of the proposed algorithm for the *K*-pairs relay-aided system in Section 6, and Section 7 concludes the paper.

*Notation.* For matrix *A*, tr⁡(*A*), rank (*A*), |*A*|, *A* ^*T*^, *A* ^*H*^, and *A* ^−1^ denote the trace, rank, determinate, transpose, conjugate transpose, and inverse of *A*, respectively. *C* ^*x*×*y*^ and *R* ^*x*×*y*^ denote the space of *x* × *y* matrices with complex and real entries. *E*(·) stands for the expectation and *D*(*A*) = diag⁡(*a*_1_,…, *a*_*n*_) are the diagonal matrix whose elements on the diagonal are *a*_1_,…, *a*_*n*_.

## 2. System Model

In this section, we propose the one-way relay system, whose key idea to structure the quasi-EVD channel is using the relay function to cancel the unitary matrices of multiple access channel (MAC) and broadcast hermitian channel (BHC).

### 2.1. Protocol Description

**Figure 1:**
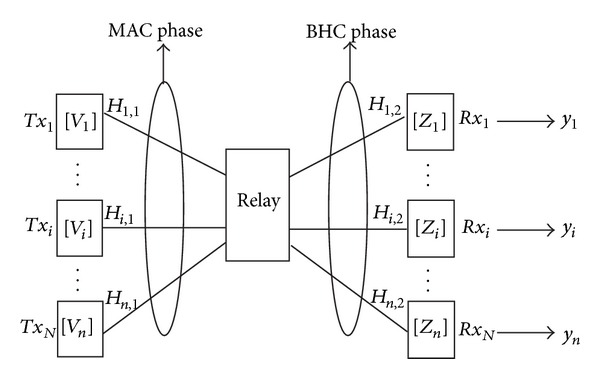
*K*-pairs single relay-aided interference alignment system.

Consider *K*-pairs interference single relay-aided system that proceeds in two phases, which are multiple access channels (MAC) and broadcast hermitian channel (BHC) as shown in Figure 1, where transmitter *Tx*_*i*_ and receiver *Rx*_*i*_ are equipped with *M* antennas, and the relay node has *NK* antennas. The channel coefficients *H*_*i*,1_ ∈ *C* ^*N*×*M*^ and *H*_*i*,2_ ∈ *C* ^*M*×*N*^ define links from the source *i* to relay and relay to the destination *i*, where *i* = 1,2,…, *K* and *M* ≤ *N* (decodable condition). The received signal at relay in the MAC phase is given by

(2) $$\begin{array}{c} r_{i}=H_{i,1}s_{i}+\overset{K}{\underset{j\neq i}{\sum }}H_{j,1}s_{j}+n_{i,1}, \end{array}$$

where *n*_*i*,1_ ~ *CN*(0, *σ*_*i*,1_^2^ *I*_*N*_) represents the additive white Gaussian noise (AWGN) vector with zero mean and variance *σ*_*i*,1_^2^. The transmitted signal form *Tx*_*i*_ to relay is obtained by the precoding matrix *V*_*i*_ ∈ *C* ^*M*×*M*^; that is, *s*_*i*_ = *V*_*i*_ *x*_*i*_ for *i* = 1,2,…, *K*, where *x*_*i*_ = [*a*_1_⋯*a*_*i*_⋯*a*_*m*_]^*T*^ is the transmitted signals form user *i* and *a*_*i*_ is date stream. The proposed precoder *V*_*i*_ can be obtained in two steps as follows: *V*_*i*_ = *V*_*i*_^*a*^ *V*_*i*_^*b*^, which will be further discussed in Section 3. The term *s*_*i*_ ∈ *C* ^*M*×1^ is subject to a power constraint, tr⁡{*E*(*s*_*i*_ *s*_*i*_^*H*^)} ≤ *P*_*i*_ with *E*(*x*_*i*_ *x*_*i*_^*H*^)≤(*P*_*i*_/*M*)*I*_*M*_, where *P*_*i*_ is the transmit power at *Tx*_*i*_.

In the BHC phase, relay sends *s*_*r*_ ∈ *C* ^*N*×1^ which is combined with the linear precoding matrix *W*_*i*_ ∈ *C* ^*N*×*N*^, to *Rx*_*i*_ as follows:

(3) $$\begin{array}{c} s_{r}=W_{i}r_{i}, \end{array}$$

where the relay precoding matrix *W*_*i*_ is subset of relay filter *W*. We assume that the maximum transmission power at relay node is *P*_*r*_; that is,

(4) $$\begin{array}{c} \mathrm{tr}\left\{W\left(\overset{K}{\underset{i=1}{\sum }}H_{i,1}V_{i,1}V_{i,1}^{H}H_{i,1}^{H}+\sigma_{i,1}^{2}I_{N}\right)W^{H}\right\}\leq P_{r}, \end{array}$$

where we have used the assumption that the source signals and the relay noise are independent with each other. Then, the relay broadcasts *s*_*r*_ to the destination nodes and the received signals at *Rx*_*i*_ can be written as

(5) $$\begin{array}{c} y_{i}=H_{i,2}s_{r}+n_{i,2}, \end{array}$$

where *n*_*i*,2_ denotes the additive noise vector at *Rx*_*i*_ with *n*_*i*,1_ ~ *CN*(0, *σ*_*i*,2_^2^ *I*_*M*_). Due to the received signal given by (5), the destination can detect the message by the MMSE criterion or

(6) $$\begin{array}{c} \varepsilon_{i}=\arg\min E\left\{{||Z_{i}^{H}y_{i}-x_{i}||}^{2}\right\}, \end{array}$$

where *Z*_*i*_ is an *M* × *M* linear decode matrix at *Rx*_*i*_.

### 2.2. Quasi-EVD and Global CSIT

We assume that the global channel state information (CSI) and the designed precoding matrices are perfectly known at all the nodes; thus, the channel coefficient can be denoted as SVD decomposition or Hermitian of SVD. In our proposed system, the channel matrices may be defined as follows:

- MAC phase: *H*_*i*,1_ = *U*_*i*,1_^*H*^Σ_*i*,1_Λ_*i*,1_,
- BHC phase: *H*_*i*,2_ = Λ_*i*,2_^*H*^Σ_*i*,2_^*H*^ *U*_*i*,2_,

where (*U*_*i*,1_, *U*_*i*,2_) ∈ *C* ^*N*×*N*^ and (Λ_*i*,1_, Λ_*i*,2_) ∈ *C* ^*M*×*M*^ are unitary matrices. Σ_*i*,1_ = [diag⁡(*λ*_1,1_,…,*λ*_*m*,1_) 0_(*N*−*M*)×*M*_]^*T*^ ∈ *C* ^*N*×*M*^ and Σ_*i*,2_ = [diag⁡(*λ*_1,2_,…,*λ*_*m*,2_) 0_(*N*−*M*)×*M*_]^*T*^ ∈ *C* ^*N*×*M*^ are eigen value matrices, where *λ*_*i*,1_ is the element of eigenvalues.

In addition, we propose the channel gain matrix which has its singular value matrix in its middle as well as its eigen matrix and unitary matrix in its right or left side appropriately, which results in the new diagonal matrix. This kind of structure is called quasi-EVD. Firstly, we show a result which is helpful to define the quasi-EVD equivalent channel as follows:

(7) Σi,2H·Σi,1=diag⁡(λ1,2∗·λ1,1,…,λm,2∗·λm,1)=diag⁡(λ1,2,1,…,λ1,2,m)=Σi,i2,

where *λ*_*a*,*b*,*i*_ = *λ*_*b*,i_* · *λ*_*a*,*i*_.

First, we proceed by reviewing the feasibility conditions of interference alignment and cancellation. Next, we turn to structure of the quasi-EVD diagonal channel and the problem of the optimization of the precoders and MSE detectors.

## 3. Optimal Filters Design and CD-BD Algorithm

### 3.1. Interference Alignment and Cancellation

As shown in [15], the IA scheme is a linear precoding technique to align interference in reduced dimensional signal subspace at each receiver. The feasibility conditions for MIMO interference channel (IC) consist of the one interference-free constraint and a signal space rank constraint. The perfect IA requirements for all *k* are

(8a) $$\begin{array}{c} U_{j}^{H}H_{j}V_{j}=0,\;\forall j\neq n, \end{array}$$

(8b) $$\begin{array}{c} \mathrm{rank}\left(U_{i}^{H}H_{i}V_{i}\right)=d_{i},\;\forall i\in \left\{1,2,\ldots ,K\right\}. \end{array}$$

An efficient distributed algorithm to find matrices *U*_*j*_ and *V*_*j*_ are derived in [16] by using the channel reciprocity. The condition (8a) guarantees that all the interfering signals at destination *j* ∈ *K* are aligned in a subspace of *N*_*k*_ − *d*_*i*_ dimensions and can be zero-forced by *U*_*j*_. Condition (8b) guarantees that destination *Rx*_*i*_ is able to decode all *d*_*i*_ intended data streams successfully. If conditions (8a) and (8b) are satisfied, then the effective channel is free from interference; the structure is feasible for the given DOF *d*_*i*_.

### 3.2. Effective Equivalent Diagonal Channel

Due to the SVD of channel in Section 2, the equivalent channel for the total system can be described as

(9) Hi=Hi,2WiHi,1=Λi,2HΣi,2HUi,2WiUi,1HΣi,1Λi,1,

where *W*_*i*_ ∈ *C* ^*N*×*N*^ is the relay precoding matrix. To eliminate the quasi-EVD channel, we adopt the relay precoding matrix defined as

(10) $$\begin{array}{c} W_{i}=U_{i,2}^{H}\times U_{i,1}. \end{array}$$

If *V*_*i*_ has full rank, *U*_*i*_^*H*^ are also with full rank. It implies that both pseudoinverses of *V*_*i*_ and *U*_*i*_^*H*^ exist. In order to get the optimal leakage interference, the relay filter should satisfy the constraint

(11) $$\begin{array}{c} W_{i}^{H}W_{i}=I_{N}. \end{array}$$

By substituting (10) into (11), the above-mentioned equation can be written as

(12) WiHWi=(Ui,2H×Ui,1)H(Ui,2H×Ui,1)=IN.

Obviously, the relay function *W*_*i*_ results in optimal leakage interference condition. In order to achieve the optimal leakage interference, it should satisfy the constraint as follows:

(13) $$\begin{array}{c} \min\left(W_{i}^{H}A_{i}W_{i}\right)=0, \end{array}$$

where *A*_*i*_ = *Z*_*i*_^*H*^ *P*_*r*_ *Z*_*i*_, *P*_*r*_ is the relay power constraint shown in (4). Therefore, when interference alignment is feasible, the objective function in (13) can be minimized. By using relay function *W*_*i*_ and (7), we may structure a quasi-EVD channel as

(14) Hi=Λi,2HΣi,2HΣi,1Λi,1=Λi,2HΣi,i2Λi,1.

**Figure 2:**
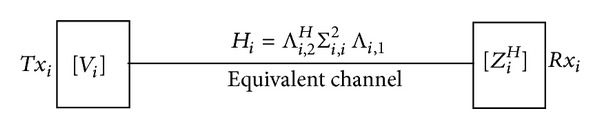
Equivalent quasi-EVD channel for relay-aided system.

Subsequently, this efficient channel for the pair of user *i* in total system can be shown in Figure 2.

Therefore, span⁡(*Z*_*i*_^*H*^ *H*_*i*_ *V*_*i*_) constitutes the useful signal space in which it is expected to observe all symbols transmitted by user *i*, while span⁡(*Z*_*j*_^*H*^ *H*_*j*_ *V*_*j*_)_*j*≠*i*_ is the space where all interference is observed. In addition, to make the leakage interference zero, the relaying function can be inserted at the relay.

The total interference leakage at the destination is given by [17]

(15) $$\begin{array}{c} \Omega_{i,2}=\mathrm{tr}\left\{Z_{i}^{H}P_{r}Z_{i}\right\}, \end{array}$$

where *P*_*r*_ is the power constraint shown in (4). Based on equivalent channel, (15) can be rewritten as

(16) $$\begin{array}{c} \Omega_{i,1}=\mathrm{tr}\left\{V_{i}^{H}{\tilde{P}}_{i}V_{i}\right\},\\ {\tilde{P}}_{i}=\overset{K}{\underset{i=1}{\sum }}\mathrm{tr}\left\{\frac{1}{d_{i}}V_{i}^{H}H_{i}^{H}Z_{i}Z_{i}^{H}H_{i}V_{i}\right\}. \end{array}$$

For the perfect interference alignment, the leakage interference should be zero, which means that *Ω*_*i*,1_ = *Ω*_*i*,2_ = 0. This equation is equivalent to the zero-forcing at *Rx*_*i*_ which is elegantly employed to achieve a good performance in the proposed scheme. The channel state information is perfectly known at every node; the optimization problem in (15) can be written as

(17) min⁡Vi,ZiH E{||ZiHyi−xi||2}s.t.  tr⁡{W(∑i=1KHi,1ViVi,1HHi,1H+σi,12IN)WH}≤Pr,

where *P*_*r*_ is the transmit power at relay. It shows that the optimization problem contains only *V*_*i*_ and *Z*_*i*_; we will further discuss details in next section.

### 3.3. Global Optimal Precoder and Detector Design

The proposed optimal precoder design involves two steps, that is, MMSE detector design at destination and optimal precoding design at transmitter. It contains two phases as follows.

#### 3.3.1. MMSE Detector Design

For the above-mentioned parameters, the sum of leakage interference can be reshaped as

(18) $$\begin{array}{c} \overset{K}{\underset{i=1}{\sum }}\Omega_{r}=\overset{K}{\underset{i=1}{\sum }}\mathrm{tr}\left\{Z_{i}^{H}P_{i}Z_{i}\right\},\; \end{array}$$

and it may be given as follows by denoting that *Q*_*i*_ = *H*_*i*,2_ *W*_*i*_ *H*_*i*,1_ *V*_*i*_:

(19) $$\begin{array}{c} Z_{i}^{\text{opt}}=Q_{i}^{H}{\left(Q_{i}Q_{i}^{H}+\sigma_{i,1}^{2}\Sigma_{i,2}^{2}+\sigma_{i,2}^{2}I_{M}\right)}^{-1},\;i=1,\ldots ,K \end{array}$$

which is the optimal MMSE decoder design proved in Appendix A. Therefore, the minimum *Ω*_*i*_ is equivalent to sum of *d*_*i*_ least dominant eigenvalues of *P*_*i*_.

#### 3.3.2. Optimal Precoding Design and Iterative Algorithm

Based on MMSE detector *Z*_*i*_^opt^, precoding matrices at source nodes should be collaboratively designed. To simply discuss the optimization problem, we assume that the noises are with same variance; that is, *σ*_*i*,1_ = *σ*_*i*,2_ = *σ*_*i*_. By using optimal MSE detector design shown in (19), the MSE matrix of the signal waveform estimation at receiver can be denoted as $\varepsilon_{i}=\left[\left({\tilde{x}}_{i}-x_{i}\right){\left({\tilde{x}}_{i}-x_{i}\right)}^{H}\right]$ or

(20) min⁡Vi εi=tr⁡{[IM+1σi2QiΨi−1QiH]−1}s.t. tr⁡{W(∑i=1KHi,1ViVi,1HHi,1H+σi,12IN)WH}≤Pr,

where Ψ_*i*_ = *I*_*M*_ + *H*_*i*,2_ *H*_*i*,2_^*H*^.

**Lemma 1 .**

The optimal precoding matrices *V*_*i*_^*b*^ design is a convex optimization in high-SNR region. For proof see Appendix B.

By applying the MMSE inversion to the combined channel matrix, we have

(21) Hmse†=HH(HHH+αI)−1=[H1,mse,H2,mse,…,HK,mse],

where *H* is the combined equivalent channel matrix; that is, *H* = [*H*_1_^*T*^,*H*_2_^*T*^,…,*H*_*K*_^*T*^]^*T*^ ∈ *C* ^*KM*×*KM*^ and *α* is the regularization factor. Considering a high-SNR case, it can be shown that *α* approaches zero and we have *HH*_mse_^†^ ≈ *I*_*KM*_. This means the off diagonal block matrices of *HH*_mse_^†^ converge to zero with high SNR. In addition, we exclude the *i*th pair user's channel matrices and define ${\overset{-}{H}}_{i,1}$ and ${\overset{-}{H}}_{i,2}$ as

(22) H−i,1=[H1,1T,…,Hi−1,1T,Hi+1,1T,…,HM,1T]T∈C(K−1)N×K(M−1),H−i,2=[H1,2T,…,Hi−1,2T,Hi+1,2T,…,HM,2T]T∈CK(M−1)×(K−1)N.

Thus, the equivalent excluded channel may be denoted as

(23) $$\begin{array}{c} {\overset{-}{H}}_{i}={\overset{-}{H}}_{i,1}{\overset{-}{W}}_{i}{\overset{-}{H}}_{i,2}\in C^{K(M-1)\times K(M-1)}. \end{array}$$

Obviously, the matrix *H*_*i*,mse_ is approximately in the null space of ${\overset{-}{H}}_{i}$ which can be expressed as

(24) $$\begin{array}{c} {\overset{-}{H}}_{i}H_{i,\text{mse}}\approx 0. \end{array}$$

Considering the SVD of *H*_*i*,mse_ = *U*_*i*,mse_Σ_*i*,mse_Λ_*i*,mse_, we have

(25) $$\begin{array}{c} {\overset{-}{H}}_{i}H_{i,\text{mse}}={\overset{-}{H}}_{i}U_{i,\text{mse}}\Sigma_{i,\text{mse}}\Lambda_{i,\text{mse}}\approx 0, \end{array}$$

where *U*_*i*,mse_ and Λ_*i*,mse_ are unitary matrices and Σ_*i*,mse_ is eigen value matrix. Since *U*_*i*,mse_ and Λ_*i*,mse_ are invertible, we have

(26) $$\begin{array}{c} {\overset{-}{H}}_{i}\Sigma_{i,\text{mse}}\approx 0. \end{array}$$

Thus, Σ_*i*,mse_ satisfies the BD constraint to balance the interference and the noise term. Therefore, the first step precoding design is completed with result *V*_*i*_^*a*^ = Σ_*i*,mse_. On the other hand, the interference generated to the other users is determined by ${\overset{-}{H}}_{i}V_{i}^{a}$. Thus, the final precoder for user *i* may be obtained as

(27) $$\begin{array}{c} V_{i}=V_{i}^{a}V_{i}^{b}=\Sigma_{i,\text{mse}}\Lambda_{i,1}^{H}B_{i}^{1/2}L_{i}. \end{array}$$

After the precoding process, the MU-MIMO channel is decoupled into a set of *K* parallel independent SU-MIMO channels by the BD precoding. In order to decode the desired signals at the corresponding receivers, the following constraints should be satisfied [9]:

(28) $$\begin{array}{c} \mathrm{span}\left(H_{m,n}V_{m}\right)=\mathrm{span}\left(H_{j,n}V_{j}\right),\;\forall m\neq n\neq j, \end{array}$$

where the precoder *V*_*m*_ is subject to the signal space. We can optimize the precoder matrix tailored to individual rate. Consequently, the total leakage interference is

(29) $$\begin{array}{c} \overset{K}{\underset{i=1}{\sum }}\Omega_{i,1}=\overset{K}{\underset{i=1}{\sum }}\mathrm{tr}\left\{V_{i}^{H}{\tilde{P}}_{i}V_{i}\right\}. \end{array}$$

As the variance of noises *σ*_*i*,1_ and *σ*_*i*,2_ is small enough in the wireless systems, the convexity can be ensured by substituting (10) and (27) into (29). While it is hard to derive a closed-form solution for (29), it can be efficiently solved using the optimal package provided in [18]. Therefore, the minimum *Ω*_*i*_ is equal to the sum of the *d*_*i*_ least dominant eigenvalues of ${\tilde{P}}_{i}$; therefore, the optimal precoder and decoder design are completed.

The proposed relay-aided interference alignment algorithm is given in Algorithm 1. By employing the minimization technique, it can iteratively update the coding vectors at transmitters, the zero-forcing vectors at receivers, and relaying function at relay to minimize the total leakage interference.

## 4. Performance Analysis

In this section, we carry out an analysis of the performance of proposed system. We consider a performance analysis in terms of BER, achievable sum of rate (ASR).

For the RBD precoding [13], the residual interference ${\overset{-}{H}}_{i}V_{i}^{a(\text{RBD})}$ is not zero between the users which is the solution in high-SNR region shown as follows:

(30) $$\begin{array}{c} \left({\overset{-}{H}}_{i}V_{i}^{a(\text{RBD})}\right){\left({\overset{-}{H}}_{i}V_{i}^{a(\text{RBD})}\right)}^{H}\approx I_{M}. \end{array}$$

By comparing (26) and (30), we can see that the impact of our proposed precoding would be smaller than that of the conventional RBD precoding algorithm.

Assuming that there exist intersections between desired signal channel and interference signal channel, the following equation will be satisfied:

(31) $$\begin{array}{c} \left[\begin{array}{ccc} I_{M} & -H_{i,1} & 0\\ I_{M} & 0 & -H_{j,1} \end{array}\right]\left[\begin{array}{c} x_{i}\\ V_{i}\\ V_{j} \end{array}\right]=0, \end{array}$$

where *x*_*i*_ is the transmitted signals from user *i*. After spanned interference signals into one dimension, we can full cancel them [19]. Therefore, the observations at the relay in (2) can yield

(32) $$\begin{array}{c} r_{i}=H_{i,1}V_{i}x_{i}+n_{i,1}, \end{array}$$

where *H*_*i*,1_ *V*_*i*_ denote column vector of total effective MAC channel matrix with size *M* × *M*. Consequently, after the relay filter *W*, the effective propagation of total system is structured and the observations of user *i* for MMSE precoding under the high-SNR scenario can be obtained as

(33) $$\begin{array}{c} y_{i}=s_{i}+\sqrt{\eta_{1}}n_{i,1}+\sqrt{\eta_{2}}n_{i,2}. \end{array}$$

Consequently, the factor that *H*_*i*,1_ *V*_*i*_^*a*^ = *U*_*i*,1_^*a*^Σ_*i*,1_^*a*^Λ_*i*,1_^*a*^ with rank *ℵ* and *H*_*i*,*i*_ *V*_*i*_^*a*^ = *U*_*i*,*i*_^*a*^Σ_*i*,*i*_^*a*^Λ_*i*,*i*_^*a*^ with rank Γ, it is simple that the normalization factors *η*_*φ*_ and *η*_*τ*_ can be written as

(34) ηφ=||(Hi,1Via)−1si||F2=tr⁡((Σi,1a)−2sisiH)=∑φ=1ℵPφ2(λφa)2,ητ=||(Hi,iVia)−1si||F2=tr⁡((Σi,1a)−2sisiH)=∑τ=1ΓPτ2(λτa)2,

where the quantity *λ*_*φ*_^*a*^, *λ*_*τ*_^*a*^, *P*_*φ*_^2^, and *P*_*τ*_^2^ are the *φ*th singular value of Σ_*i*,1_^*a*^, *τ*th singular valve of Σ_*i*,1_^*a*^, energy of *φ*th, and *τ*th stream of *s*_*i*_, respectively. From (34), the received SNR for *l*th date of user *i* is obtained as

(35) $$\begin{array}{c} \text{SN}{\text{R}}_{l}=\frac{P_{l}}{\sigma_{n}^{2}\left(\sum_{\varphi =1}^{ℵ}P_{\varphi }^{2}{\left(\lambda_{\varphi }^{a}\right)}^{-2}+\sum_{\tau =1}^{\Gamma }P_{\tau }^{2}{\left(\lambda_{\tau }^{a}\right)}^{-2}\right)}. \end{array}$$

Then, the SR upper bound for *i*th user can be calculated as

(36) $$\begin{array}{c} C_{i}\leq \overset{\max(ℵ,\Gamma )}{\underset{l=1}{\sum }}\log\left(1+\frac{P_{i}}{\sigma_{i}^{2}\sum_{\varphi ,\tau =1}^{\max(ℵ,\Gamma )}\left(\eta_{\varphi }+\eta_{\tau }\right)}\right). \end{array}$$

It shows that *C*_*i*_ contains only normalization factors *η*_*φ*_ and *η*_*τ*_. The maximum value of *C*_*i*_ is achieved only and only if *P*_1_^2^/(*λ*_1_^*a*^)^2^ = ⋯ = *P*_*φ*_^2^/(*λ*_*φ*_^*a*^)^2^ = ⋯ = *P*_*τ*_^2^/(*λ*_*τ*_^*a*^)^2^; thus, the ASR for total system at high-SNR region can be expressed as

(37) $$\begin{array}{c} C\leq \overset{K}{\underset{i=1}{\sum }}\;\overset{\max(ℵ+\Gamma )}{\underset{i=1}{\sum }}\log\left(1+\frac{P_{l}}{2\sigma_{n}^{2}\max\left(ℵ,\Gamma \right)}\right). \end{array}$$

Therefore, the total achievable DOF for this network can be represented as the sum of DOF for each link [20]. Consider

(38) dtotal=lim⁡SNR→∞∑i=1Kdi,j=lim⁡SNR→∞∑i=1KClog⁡⁡(SNR),

where *d*_*i*,*j*_ denotes the DoF for the transmission from user *i* to user *j*.

## 5. Computational Complexity Analysis

In this section, we will compare the computational complexity of proposed scheme and prior works. We use the total number of floating point operations (PLOPs) to measure the computational complexity. According to [21], the required FLOPs of each matrix operation are described as follows:

- multiplication of *m* × *n* and *n* × *p* complex matrices: 8*mnp* − 2*mp*;
- multiplication of *m* × *n* and *n* × *m* complex matrices: 4*nm* × (*m* + 1);
- SVD of and *m* × *n*  (*m* ≤ *n*) complex matrix where only Σ is obtained: 32(*mn* ^2^ − *n* ^3^/3);
- SVD of and *m* × *n*  (*m* ≤ *n*) complex matrix where only Σ and Λ are obtained: 32(*nm* ^2^ + 2*m* ^3^);
- SVD of and *m* × *n*  (*m* ≤ *n*) complex matrix,where only *U*, Σ, and Λ are obtained: 8(4*n* ^2^ *m* + 8*nm* ^2^ + 9*m* ^3^);
- inversion of an *m* × *m* real matrix using Gauss-Jordan elimination: 2*m* ^3^ − 2*m* ^2^ + *m*;
- Cholesky factorization of an *m* × *m* complex matrix: 8*m* ^3^/3.

For the conventional RBD method [13], the authors consider a multiuser MIMO downlink precoding system with a base station communicating with *K*-users simultaneously. For the nonregenerative MIMO relay systems [14], the authors consider a 3-node MIMO relay, where multiple antennas are equipped at the source *S*, the relay *R*, and the destination *D*. We compare the required FOLPs of each precoding algorithm for proposed method, conventional RBD, and nonregenerative MIMO relay system in Tables 1, 2, and 3, respectively, where we assume that *N*_*T*_ = *N*_*R*_ and ${\overset{-}{N}}_{i}=N_{T}-N_{i}$.

For instance, the (2,2, 2) × 6 case denote a system with user *K* = 3, each user with *N*_*i*_ = 2 antennas, and total transmit antennas is *N*_*T*_ = 6. The required FLOPs of the proposed method, conventional RBD, and the nonregenerative MIMO relay system are counted as 34638, 40824, and 45306, respectively. From the results, we can see that the reduction in the number of FLOPs and the proposed method precoding are 15.15% and 23.55% as compared to the conventional RBD and the nonregenerative MIMO relay systems. Thus, the proposed algorithms exhibit lower complexity than the conventional RBD and the nonregenerative MIMO relay system approaches, and the complexity advantage grows as *N*_*i*_, *N*_*T*_, and *K* increase.

## 6. Simulation Results

In this section, we show the performance of the proposed scheme in terms of the computation complexity, achievable sum-of-rate (ASR), and BER performance with some simulation results.

**Figure 3:**
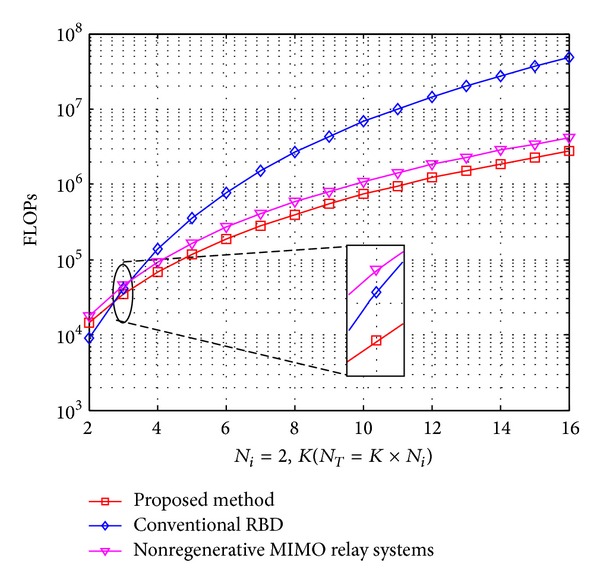
The complexity comparisons for required FLOPs versus the number of the users *K*.

**Figure 4:**
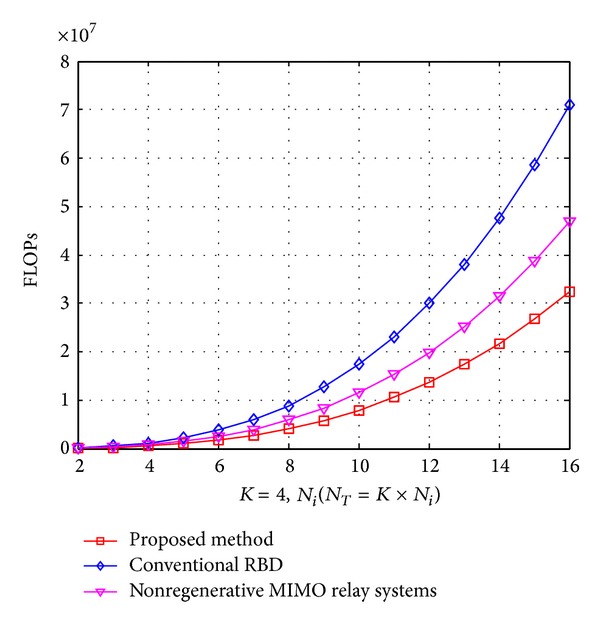
The complexity comparisons for required FLOPs versus the number of the receive antennas *N*_*i*_ for each user.

Using Tables 1, 2, and 3, we give the calculated results of FLOPs of the alternative methods in Figures 3 and 4. In the first comparison shown in Figure 3, we consider the case that *N*_*T*_ = *K* × *N*_*i*_. We set *N*_*i*_ = 2 and express the computation cost as a function of *K*.

In Figure 4, we fix user *K* = 4 and *N*_*T*_ = *K* × *N*_*i*_ while the computation cost as a function of *N*_*i*_. For conventional RBD method, the orthogonal complementary vector *V*_*k*,0_ with dimension ${\overset{-}{N}}_{i}\times N_{T}$ is obtained; it requires *K* times SVD operations and if we only want to compute *V*_*k*,0_, the computational is not efficient. In Step 5, after we got efficiency channel *H*_eff_ = *H*_*i*_ *P*_*i*_^*a*^, the second SVD operation should be carried out with dimension *R*_eff_ × *N*_*T*_, where *R*_eff_ is the rank of *H*_eff_.

For nonregenerative MIMO relay system method, to simply discuss computational complexity, only the indirect link part algorithm is shown. In Steps 1 and 2, two SVD operations are required for the channels from the source to relay and relay to the destination Two variances *H*_*i*_^*H*^ *H*_*i*_ and *H*_*j*_^*H*^ *H*_*j*_ are needed to structure *A* as shown in Step 5. Finally, SVD *A* and diagonalize *G*.

For the proposed algorithm, the second precoding matrix *V*_*i*_^*b*^ is structured by using Cholesky decomposition instead of SVD operation and the first precoding matrix *V*_*i*_^*a*^ is calculated by SVD of *H*_*i*,mse_^†^, but only eigenvalue matrices are obtained. Obviously, the proposed method shows a clear advantage in comparisons.

**Figure 5:**
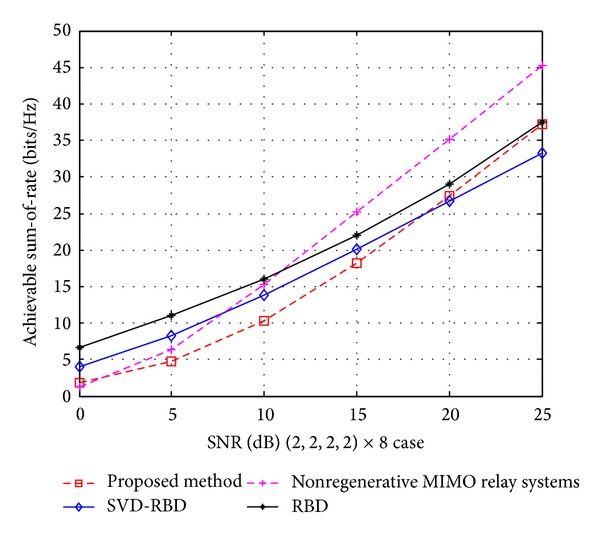
The achieved sum-of-rate of SVD-BD, RBD, nonregenerative MIMO relay systems, and proposed method for (2,2, 2,2) × 8 case.

**Figure 6:**
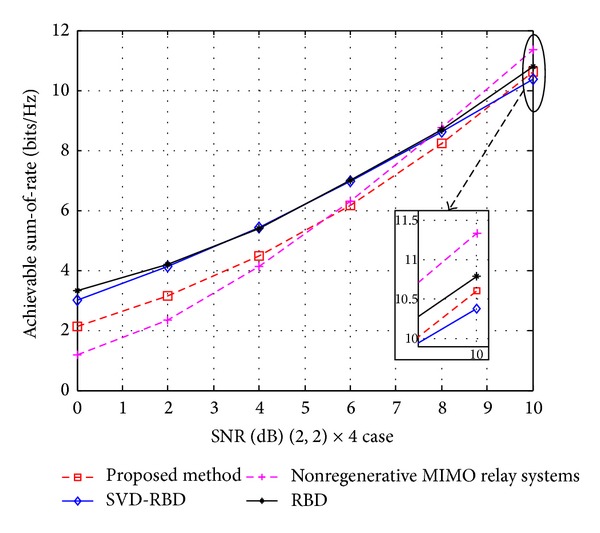
The achieved sum-of-rate of SVD-BD, RBD, nonregenerative MIMO relay systems, and proposed method for (2,2) × 4 case.

In Figures 5 and 6, we compare the sum-of-rate of various MU-MIMO schemes under full CSI known at each node. The total capacity is obtained by using [22]

(39) $$\begin{array}{c} C_{\text{sum}}=\log\left(\det\left(I+\sigma_{n}^{-2}HPP^{H}H^{H}\right)\right), \end{array}$$

and the ASR of proposed method is computed using (35), (36), and (37). Figures 5 and 6 illustrate the sum-of-rate as a function of SNR for (2,2, 2,2) × 8 and (2,2) × 4 cases, respectively.

In Figures 5 and 6, the nonregenerative MIMO relay systems show a better sum-of-rate than others at high SNRs, because its capacity includes direct links form source to the destinations and indirect links via relay. The RBD precoding with SVD provides higher ASR than BD at whole SNRs. It is clear that the ASR of our proposed precoding algorithm is lower than the BR at low SNRs, but at high-SNR regime, it is higher than SVD-RBD and almost same as RBD.

**Figure 7:**
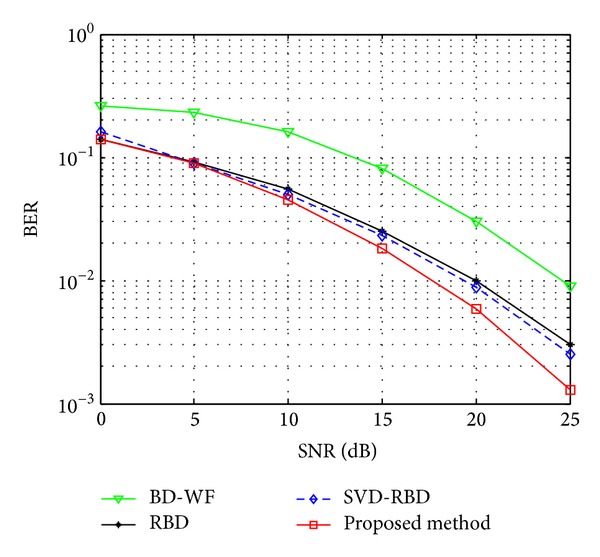
BER performance with QPSK.

In Figure 7, we compare the BER performance of BD-water filling, RBD, SVD-RBD, and proposed method, where QPSK modulation is applied. The proposed algorithm achieved better performance than existing precoding algorithms. As shown in Figure 7, the global optimal scheme in Section 3.3 is evaluated, the reason is that the precoding matrix *V*_*i*_^*a*^ restricts the interference between the users close to zero while the other precoding algorithm is *I*_*M*_. The performances significantly improve with increase of SNR.

## 7. Conclusion

In this paper, motivated by the structure of the quasi-EVD based channel in the relay-aided system, we have demonstrated a novel iterative algorithm. Our goal is to achieve the maximum sum-of-rate and the minimum leakage interference. To minimize leakage interference, we use interference alignment to minimize the overlap of the row spaces spanned by the effective channels of different users. The design of the precoding matrix presented in this paper is general, which also can target minimum BER and reduce the computational complexity. In the first step, we use the Cholesky and the singular value decomposition to design the second part of precoder and solve the optimization problem for the total system with MMSE detector. In the next step, we apply the MMSE inversion to the equivalent channel to minimize the BER, which completes the first part of the precoder design. According to the precoding processes, the MU-MIMO channel is decoupled into a set of parallel independent SU-MIMO channels. Simulation results show that the proposed algorithm outperforms the existing techniques.

## Figures and Tables

**Algorithm 1:**
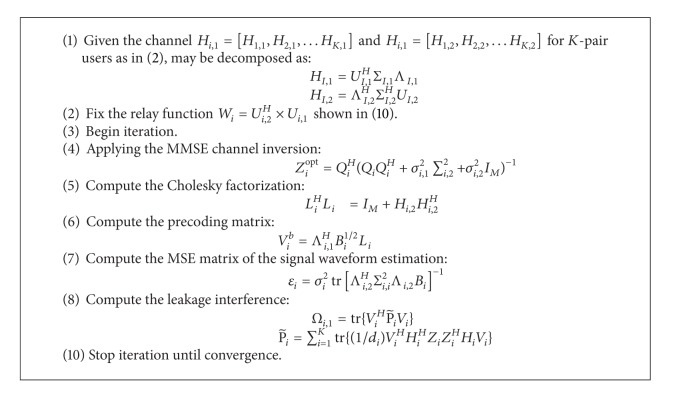
Cholesky decomposition-block diagonalization (CD-BD) algorithm.

**Table 1:** Computational complexity of proposed Algorithm 1.

Steps	Operations	FLOPs	Case (2,2, 2) × 6
1 (a)	U_*i*,1_^*H*^Σ_*i*,1_Λ_*i*,1_	8*K*(4*N*_*T*_^2^ *N*_*i*_ + 8*N*_*T*_ *N*_*i*_^2^ + 9*N*_*i*_^3^)	13248
1 (b)	Λ_*i*,2_^*H*^Σ_*i*,2_^*H*^U_*i*,2_	8*K*(4*N*_*T*_^2^ *N*_*i*_ + 8*N*_*T*_ *N*_*i*_^2^ + 9*N*_*i*_^3^)	13248
2	H_*i*,2_WH_*i*,1_	$\begin{array}{c} K\left[8N_{i}N_{T}^{2}-2N_{i}N_{T}+4N_{i}N_{T}\times \left(N_{i}+1\right)\right] \end{array}$	2088
3	L_*i*_^*H*^L_*i*_	$\begin{array}{c} 2K\left[N_{i}+2N_{T}N_{i}\times \left(N_{i}+1\right)+4N_{i}^{3}/3\right] \end{array}$	508
4	H_mse_^†^	$\begin{array}{c} 4N_{R}^{3}/3+12N_{R}^{2}N_{T}-2N_{R}^{2}-2N_{T}N_{R} \end{array}$	2736
5	H_*i*,*i*_V_*i*_^*a*^V_*i*_^*b*^	$\begin{array}{c} 8K\left[4N_{T}N_{i}^{2}-4N_{i}^{3}/3+N_{i}^{2}\left(N_{i}+1\right)\right] \end{array}$	2336
6	(*Q*_*i*_ *Q*_*i*_^*H*^ + *σ*_*i*_^2^Ψ_*i*_)^−1^	$\begin{array}{c} K\left[4N_{R}N_{i}\times \left(N_{i}+1\right)+3N_{i}+2N_{i}^{3}-2N_{i}^{2}\right] \end{array}$	474

Total			34638

**Table 2:** Computational complexity of conventional RBD [13].

Steps	Operations	FLOPs	Case (2,2, 2) × 6
1	U_*i*_^*a*^Σ_*i*_^*a*^Λ_*i*_^*aH*^	$32K(N_{T}{\overset{-}{N}}_{i}^{2}+2{\overset{-}{N}}_{i}^{3})$	21504
2	(Σ_*i*_^*a*^ ^*T*^Σ_*i*_^*a*^ + *ρ* ^2^ *I*_*T*_) ^−1/2^	$K(18N_{T}N_{i}^{2}+{\overset{-}{N}}_{i})$	336
3	V_*i*_^*a*^ *D*_*i*_^*a*^, (*D*_*i*_^*a*^ ← 2)	8*KN*_*T*_^3^	5184
4	H_*i*_P_*i*_^*a*^	*K*(8*N*_*T*_ *N*_*i*_^2^ − 2*N*_*i*_^2^)	552
5	U_*i*_^*b*^Σ_*i*_^*b*^V_*i*_^*bH*^	64*K*((9/8)*N*_*i*_^3^ + *N*_*T*_ *N*_*i*_^2^ + (1/2)*N*_*T*_^2^ *N*_*i*_)	13248

Total			40824

**Table 3:** Computational complexity of nonregenerative MIMO relay system [14].

Steps	Operations	FLOPs	Case (2,2, 2) × 6
1	U_*i*_^*a*^Σ_*i*_^*a*^Λ_*i*_^*aH*^	8*K*(4*N*_*T*_^2^ *N*_*i*_ + 8*N*_*T*_ *N*_*i*_^2^ + 9*N*_*i*_^3^)	13248
2	U_*j*_^*a*^Σ_*j*_^*a*^Λ_*j*_^*aH*^	8*K*(4*N*_*T*_^2^ *N*_*i*_ + 8*N*_*T*_ *N*_*i*_^2^ + 9*N*_*i*_^3^)	13248
3	H_*i*_^*H*^H_*i*_	4*KN*_*i*_ *N*_*T*_(*N*_*i*_ + 1)	432
4	H_*j*_^*H*^H_*j*_	4*KN*_*i*_ *N*_*T*_(*N*_*i*_ + 1)	432
5	$\begin{array}{c} H_{i}^{H}{\left[\sigma_{1}^{2}\sigma_{2}^{-2}{\left(H_{j}F\right)}^{H}H_{j}F+I\right]}^{-1}H_{i} \end{array}$	$\begin{array}{c} 2K\left(N_{i}^{3}+8N_{i}N_{T}^{2}+4N_{i}^{2}N_{T}+2N_{i}N_{T}-N_{i}^{2}+N_{i}\right) \end{array}$	4212
6	V_*A*_Λ_*A*_V_*A*_^*H*^	$\begin{array}{c} 8K\left(4N_{T}^{2}N_{i}+8N_{T}N_{i}^{2}+9N_{i}^{3}+(N_{i}/2)\right) \end{array}$	13272
7	$\mathrm{diag}(\tilde{G})$	$\begin{array}{c} K[4N_{i}N_{T}(N_{i}+1)+2N_{i}^{3}\;-2N_{i}^{2}+N_{i}] \end{array}$	462

Total			45306

## Appendices

### A. The proof of Optimal MSE

The MSE at receiver can be further expressed as

(A.1) εi=argmin⁡⁡E{||ZiHyi−xi||2}=tr⁡{(ZiH(Hi,2sr+ni,2)−xi)   ×(ZiH(Hi,2sr+ni,2)−xi)H}=tr⁡(ZiHHi,2WiHi,1PPHHi,1HWiHHi,2HZi−ZiHHi,2WiHi,1P   −PHi,1HWiHi,2HZi+σi,12ZiHHi,2WiWiHHi,2HZi   +σi,22ZiZiH+IM),

where we have assumed that the signals and noise are independent with each other. Based on (10), the derivation of optimal MSE detection matrix *Z*_*i*_^opt^ is equivalent to solving the following equation:

(A.2) $$\begin{array}{c} \frac{\partial {\tilde{x}}_{i}}{\partial Z_{i}}=2Z_{i}^{H}QQ^{H}+2\sigma_{i,2}^{2}Z_{i}^{H}+2\sigma_{i,1}^{2}H_{i,2}H_{i,2}^{H}Z_{i}^{H}-2Q^{H}=0, \end{array}$$

where *Q*_*i*_ = *H*_*i*,2_ *W*_*i*_ *H*_*i*,1_ *V*_*i*_. To evaluate the efforts of the result, tr⁡(*H*_*i*,2_ *H*_*i*,2_^*H*^) can be further developed as follows by applying singular value decomposition (SVD) shown in Section 2.2 on BCH channel:

(A.3) tr⁡(Hi,2Hi,2H)=tr⁡(Λi,2HΣi,2HUi,2Ui,2HΣi,2Λi,2)=∑i=1M|λi,2|2,

where *λ*_*i*,2_ is the eigenvalues of *H*_*i*,2_. Then, the closed-form expression of *Z*_*i*_^opt^ can be obtained, which can be expressed as

(A.4) $$\begin{array}{c} Z_{i}^{\text{opt}}=Q_{i}^{H}{\left(Q_{i}Q_{i}^{H}+\sigma_{i,1}^{2}\Sigma_{i,2}^{2}+\sigma_{i,2}^{2}I_{M}\right)}^{-1}; \end{array}$$

this completes the proof.

### B. The proof of Lemma 1

Proof In the high-SNR region, the objective function *ε*_*i*_ can be expressed approximately as

(B.1) $$\begin{array}{c} \varepsilon_{i}=\mathrm{tr}{\left[\frac{1}{\sigma_{i}^{2}}Q_{i}\Psi_{i}^{-1}Q_{i}^{H}\right]}^{-1}. \end{array}$$

Since the matrix Ψ_*i*_ in the above function is Hermitian and positive definite, we can decompose this matrix using Cholesky factorization as

(B.2) $$\begin{array}{c} \Psi_{i}=I_{M}+H_{i,2}H_{i,2}^{H}=L_{i}^{H}L_{i}, \end{array}$$

where *L*_*i*_ is an *M* × *M* upper triangular matrix. Thus, the MSE *ε*_*i*_ can be rewritten as

(B.3) $$\begin{array}{c} \varepsilon_{i}=\mathrm{tr}{\left[\frac{1}{\sigma_{i}^{2}}Q_{i}L_{i}^{-1}{\left(L_{i}^{H}\right)}^{-1}Q_{i}^{H}\right]}^{-1}. \end{array}$$

Using equivalent channel *H*_*i*_ = *H*_*i*,2_ *W*_*i*_ *H*_*i*,1_ = Λ_*i*,2_^*H*^Σ_*i*,*i*_^2^Λ_*i*,1_, *Q*_*i*_ can be denoted as Λ_*i*,2_^*H*^Σ_*i*,*i*_^2^Λ_*i*,1_ *V*_*i*_, replace *Q*_*i*_ into (B.1), we can rewrite (B.1) as

(B.4) $$\begin{array}{c} \varepsilon_{i}=\mathrm{tr}{\left[\frac{1}{\sigma_{i}^{2}}\Lambda_{i,2}^{H}\Sigma_{i,i}^{2}\Lambda_{i,1}V_{i}L_{i}^{-1}{\left(L_{i}^{H}\right)}^{-1}V_{i}^{H}\Lambda_{i,1}^{H}\Sigma_{i,i}^{2}\Lambda_{i,2}\right]}^{-1}. \end{array}$$

When MSE of the signal waveform estimation is adopted as the optimal problem in (20) which is solved in [23], the precoding matrices at source can be designed as

(B.5) $$\begin{array}{c} V_{i}^{b}=\Lambda_{i,1}^{H}B_{i}^{1/2}L_{i}, \end{array}$$

where *B*_*i*_ ∈ *C* ^*M*×*M*^ is a diagonal matrix as power constraint, *A* = *V*_*i*_ *V*_*i*_^*H*^ = Λ_*i*,1_^*H*^ *B*_*i*_^1/2^ *L*_*i*_ *L*_*i*_^*H*^ *B*_*i*_^1/2^Λ_*i*,1_ = *B*_*i*_. Replacing the precoding matrix *V*_*i*_ into *ε*_*i*_, the optimization problem is obtained as

(B.6) εi=σi2tr⁡[Λi,2HΣi,i2Λi,2Bi]−1=σi2∑i=1M ∑i=1M|λ1,2,i|−2bi,

where *λ*_1,2,*i*_ is structured shown in (7); that is, *λ*_1,2,*i*_ = *λ*_1,*i*_ · *λ*_2,*i*_ and *b*_*i*_ is the diagonal elements of matrices *B*_*i*_. Similar to Lemma 2 in [24], *ε*_*i*_ is convex if and only if *ε*_*i*_ = *h*(*A*(*λ*_1,2,1_,…, *λ*_1,2,*M*_)) is convex and nonincreasing with *A* and *A* = *g*(*V*_*i*_) is a concave function of *V*_*i*_. The Hessian matrices of *V*_*i*_ is ∇_*V*_*i*_,*V*_*i*_^*H*^_ *A* = 0 which is seminegative definite; it holds that *g*(*V*_*i*_) is a concave function of *V*_*i*_. Thus, Lemma 1 has been proven.
